# Breadth of Coverage, Ease of Use, and Quality of Mobile Point-of-Care Tool Information Summaries: An Evaluation

**DOI:** 10.2196/mhealth.6189

**Published:** 2016-10-12

**Authors:** Emily Johnson, Vamsi K Emani, Jinma Ren

**Affiliations:** ^1^ University of Illinois at Chicago Library Library of the Health Sciences - Peoria University of Illinois at Chicago Peoria, IL United States; ^2^ Department of Internal Medicine University of Illinois College of Medicine at Peoria Peoria, IL United States; ^3^ Center for Outcomes Research Department of Medicine University of Illinois College of Medicine at Peoria Peoria, IL United States

**Keywords:** mHealth, mobile health, mobile app, assessment, internal medicine, point-of-care tools

## Abstract

**Background:**

With advances in mobile technology, accessibility of clinical resources at the point of care has increased.

**Objective:**

The objective of this research was to identify if six selected mobile point-of-care tools meet the needs of clinicians in internal medicine. Point-of-care tools were evaluated for breadth of coverage, ease of use, and quality.

**Methods:**

Six point-of-care tools were evaluated utilizing four different devices (two smartphones and two tablets). Breadth of coverage was measured using select *International Classification of Diseases, Ninth Revision*, codes if information on summary, etiology, pathophysiology, clinical manifestations, diagnosis, treatment, and prognosis was provided. Quality measures included treatment and diagnostic inline references and individual and application time stamping. Ease of use covered search within topic, table of contents, scrolling, affordance, connectivity, and personal accounts. Analysis of variance based on the rank of score was used.

**Results:**

Breadth of coverage was similar among Medscape (mean 6.88), Uptodate (mean 6.51), DynaMedPlus (mean 6.46), and EvidencePlus (mean 6.41) (*P*>.05) with DynaMed (mean 5.53) and Epocrates (mean 6.12) scoring significantly lower (*P*<.05). Ease of use had DynaMedPlus with the highest score, and EvidencePlus was lowest (6.0 vs 4.0, respectively, *P*<.05). For quality, reviewers rated the same score (4.00) for all tools except for Medscape, which was rated lower (*P*<.05).

**Conclusions:**

For breadth of coverage, most point-of-care tools were similar with the exception of DynaMed. For ease of use, only UpToDate and DynaMedPlus allow for search within a topic. All point-of-care tools have remote access with the exception of UpToDate and Essential Evidence Plus. All tools except Medscape covered criteria for quality evaluation. Overall, there was no significant difference between the point-of-care tools with regard to coverage on common topics used by internal medicine clinicians. Selection of point-of-care tools is highly dependent on individual preference based on ease of use and cost of the application.

## Introduction

### State of Mobile Use in Public and Health Care

For the last decade, a surge of technology has had an increasing influence on human life. Over two-thirds of American adults now own a smartphone of some kind and mobile now represents almost two out of three digital media minutes time [1,2]. The field of medicine is not an exception in this adoption of new technology, changing the practice from electronic health records to robotic surgeries.

A tool that is becoming an integral part of medical practice is the mobile device, a telecommunication device running an operating system with the ability to customize by accessing apps, word processing, and Internet connectivity. Common terms for the mobile device include smartphone and tablet, frequently adopting the branded name of the device. Use of smartphones by physicians has plateaued to 80% to 85%, and adoption of tablets has risen to 76% in 2014, according the group Manhattan Research [3]. In 2014, another survey reported over half of US hospitals use mobile devices in their facilities either by offering device programs in their institution or using a bring-your-own-device model [4].

Through the prevalent use of the mobile device, clinical professions in the health sciences now have immediate access to essential information for patient treatment, which has shown improvement in decision making and reduced medical errors, improved communication between medical staff, and enhanced telemedicine capability [5,6].

### Definition of a Point-of-Care Tool

In the everyday practice of medicine, physicians are increasingly relying on access to electronic medical resources to make clinical decisions, replacing the condensed pocket reference texts. Physicians often utilize online point-of-care tools, a resource with immediate access to filtered summaries providing current recommendations for the management of medical problems [7,8]. These resources are available globally and many are accessible via a personal subscription or an institutional license on a desktop or laptop computer.

With the emergence of mobile devices, the market of mobile health applications has grown to over 100,000 apps in the two leading operating systems, iOS and Android [9]. Point-of-care tools were no exception to this expansion of this market, including many entering the marketplace since 2008. Medical reference and diagnostic medical apps make up about 18% of the mobile health (mHealth) app market share [9]. Point-of-care tools are increasingly accessible via mobile native apps, Web apps, or both.

### Evaluation of Point-of-Care Tools on Mobile Devices

A literature review was conducted to identify articles evaluating mobile point-of-care tools with a focus on breadth of coverage, quality of evidence, and mobile ease of use design [8,10-18]. Studies were identified evaluating the quality and breadth of content of the full online Web versions of these point-of-care tools. Prorok et al and Banzi et al both evaluated the quality and breadth of coverage, determining no single point-of-care tool was ideal and clinicians should not rely on one single product [8,12].

The evaluation of mobile medical apps is in its infancy, with much of the evidence stemming from evaluation from handheld personal digital assistant devices [6,19-23]. Ease of use and mobile interface design evaluation criteria are often too general, complex, or specific for health-related mobile resources [22]. The Healthcare Information and Management Systems Society (HIMSS) has compiled guidelines to evaluate mHealth, including criteria for efficiency and platform optimization, but did not include methods of evaluation of information [24]. Stoyanov et al published the Mobile App Rating Scale for consumer health applications during the data collection of this study and covers similar criteria regarding ease of use on navigation and gestural design but did not have the detailed criteria for rating clinical point-of-care tools [22].

### The Goal of This Research Study

The research has, to date, focused on evaluating the breadth of content and quality measures and timeliness of the online versions of point-of-care tools and the user experience within mobile application design; no study has reviewed multiple mobile point-of-care tools and devices using the same evaluation criteria. With increased usage of mobile technology, there is a need to evaluate measures of quality and breadth of content of mobile versions of point-of-care tools. In addition, this research study will include an evaluation of ease of use which may affect the way these point-of-care tool appls are used and could impact patient care.

### Objective

The objective is to evaluate if six selected mobile point-of-care tools meet the defined criteria for breadth of coverage, ease of use, and quality on the different mobile devices and operating systems.

## Methods

### Selection of Point-of-Care Tools

Point-of-care tools were created for use by health care providers and focused on providing answers to clinical questions. Selection for evaluation used the following criteria: English language only, availability in the United States, and accessibility via a mobile device using either a website or an app. If the point-of-care tool was an app, it needed to be accessible on both iOS and Android devices, and it needed to identify as an evidence-based summary product with topics of internal medicine.

The researchers reviewed past evaluations of point-of-care tools within the literature [8,10-18] and reviewed the app store categories *medical* and *medicine* for Android and iOS devices; if there was no app, researchers verified there was a mobile-enabled website. Based on the defined criteria, the researchers selected six point-of-care tools: DynaMed, DynaMedPlus, Epocrates, Essential Evidence Plus, Medscape, and UpToDate. While DynaMed and DynaMedPlus are created by the same vendor, they provided unique content summaries and user interfaces, so both tools were included in the evaluation.

Accessibility of these resources was either provided by free access (Medscape), paid individual subscription (Epocrates and DynaMedPlus), or made available with a paid institutional subscription from the University of Illinois at Chicago University Library (DynaMed, Essential Evidence Plus, and UptoDate) for complete access of the content.

### Selection of Devices

Four different mobile devices were used in the analysis: two smartphones, the iPhone 5S and Moto X second generation, and two tablets, the iPad Mini 2 and Samsung Galaxy tablet. One investigator utilized the iOS operating system on the iPhone and iPad and the other investigator worked with the Android operating system on the Moto X and Samsung tablet. The selections of these devices were based on availability to the researchers and popularity in the medical field [9].

### Interrater Reliability

Prior to the full assessment of the criteria, the interrater reliability was examined in a separate dataset, in which two reviewers rated four point-of-care tools, (ACP SmartMedicine, DynaMed, Essential Evidence Plus, and UptoDate) for three *International Classification of Diseases, Ninth Revision (ICD-9)*, diagnosis codes for breadth of coverage, ease of use, and quality on four mobile devices independently.

### Breadth of Coverage Assessment

To evaluate the breadth of coverage, ICD-9 billing codes were culled from the setting of a teaching medical center with 609 beds and a level I trauma center in the state of Illinois. A total of 30 codes for the most billed from January to July 2015 were selected from the inpatient and outpatient settings. From the 30 codes, specialties other than general internal medicine were excluded and any duplicated codes utilized both in inpatient and outpatient treatment were removed, bringing the count to 17 total codes used in the evaluation (Multimedia Appendix 1).

The definition of breath of coverage was modified from prior studies [8,12]; each medical topic needed to include information consisting of a summary of the topic, etiology, pathophysiology, clinical manifestations, diagnosis, treatment, and prognosis (Multimedia Appendix 2). These topics cover the most consistently needed information for a patient encounter. If any of the information was incomplete or missing from the medical topic, it was deemed to be uncovered [8,12].

Two researchers independently entered text searches of the ICD-9 diagnosis code into the search engine of each point-of-care tool on the four devices. If searches did not return relevant results, synonyms for the code were utilized. When content was retrieved, the researcher reviewed the content to meet the breadth of coverage topics. Any disagreements on search terms and content topics was resolved by consensus.

### Quality Assessment

Methodology to assess quality of point-of-care tools was adapted from a previous study by Prorok et al^8^ and modified for a mobile point-of-care tool investigation. Quality measures included inline references for treatment and inline references for diagnostic recommendations (Multimedia Appendix 3). Time stamping of individual content and the app platform were included in the quality measures to evaluate the currency of the content and application. The app version number was included within the time stamping of the app platform criteria. The app’s update logs were able to verify the date of that version of the app. The two researchers independently verified the quality measures in all of the point-of-care tools on the four devices, and any disagreements were resolved by consensus.

The other editorial quality measures set by Prorok, such as reviewing policies on finding new evidence, grading recommendations, and updating materials, were surveyed in the initial screening of the mobile point-of-care tools and were consistent with the previous evaluation. The investigators focused on the quality measures that could have presented differently on the mobile platform.

### Ease of Use Assessment

The scale defined to report on ease of use of the point-of-care tools on a mobile device was developed utilizing design aspects by HIMSS and Nielsen Norman Group user experience criteria [24,25]. The ease of use for each point-of-care tool application was based on the following defined factors:

- Search within topic: user can use a search feature within a topic to find information.
- Table of contents: topic contains appropriate table of contents for easy navigation.
- Scrolling: appropriate scrolling patterns were utilized within the topic for mobile applications.
- Affordance: it is made clear what items can be selected, tapped, or swiped to connect with other content.
- Connectivity: content is available via Wi-Fi or mobile data connection.
- Personal account: required personal account is easy to log in and modify settings for the point-of-care tool.

The two researchers independently verified the ease of use measures in all of the point-of-care tools on the four devices, and any disagreements were resolved by consensus.

The final version of the assessment criteria, definitions, and rating system are available with this paper (see Multimedia Appendix 4).

### Statistical Analysis

Statistical analysis was performed by SAS version 9.4 (SAS Institute Inc). Mean and standard deviation as well as histogram were applied to depict the scores of point-of-care tools. The mean for breadth of coverage was calculated using the average total score of each point-of-care tool. The means for ease of use and the quality measurement was the average total score for each mobile device. A statistical significance level of .05 was set for all hypothesis tests.

The interrater reliability was statistically calculated by using both Pearson correlation coefficient and kappa coefficient for each domain (breadth, ease of use, and quality) between two reviewers in order to determine how consistent their ratings were prior to the full dataset assessment.

Later, in the whole dataset, the researchers generated new rank scores based on the rating scores for each domain because the distribution of rating scores was extremely skewed. A general linear regression model was used to compare the rank scores among the six point-of-care tools, controlling for type of mobile devices and diagnosis codes. Bonferroni-adjusted *P* values were provided when multiple comparisons were performed (See Multimedia Appendix 5 for calculations). For clearer understanding of the results, a multiple comparison model was used. If point-of-care tools are grouped under the same letter, it indicates that the differences are not statistically significant. If the point-of-care tools are not grouped, the comparisons for that criteria are statistically significant.

## Results

### Main Findings

Each of the six selected point-of-care tools was evaluated on the breadth of coverage, quality of evidence, and ease of use on the four mobile devices. After discussion and calibration of the survey instruments through an interrater review, data were collected and evaluated between May and September 2015.

### Interrater Reliability

The two reviewers had high interrater reliability overall. The correlation coefficients were *r*=.97 (*P*<.001), *r*=.88 (*P*<.001), and *r*=.41 (*P*=.07) in the domains of breadth, ease of use, and quality, respectively. The kappa coefficients were .84 (95% CI 0.77-0.91), .76 (95% CI 0.56-0.96), and .29 (95% CI 0.02-0.55) in the domains of breath, ease of use, and quality, respectively.

### Breadth of Coverage Measure

On all devices, the breadth of coverage of selected ICD-9 codes by each point-of-care tool was scored (n=68). The result represents a mean of the scores for each tool on all the devices. The mean is average total score of breath of coverage of each tool.

The mean scores of breadth of coverage were very close among Medscape (6.88), DynaMedPlus (6.47), and Essential Evidence Plus (6.41) with *P* values of >.99 (Medscape vs DynaMedPlus), 0.259 (Medscape vs Essential Evidence Plus), and >.99 (DynaMedPlus vs Essential Evidence Plus). As shown in Table 1, DynaMed had lowest score of breadth among the tools (*P*<.01), and Epocrates also had a relative lower score of breadth compared to the top four tools (*P*<.01).

The researchers did not find any statistical difference in the total score attained by each point-of-care tool on different electronic devices with different operating systems. There was also no statistical difference noted between smartphone application and tablet application for the same point-of-care tool.

### Ease of Use Measure

Ease of use was scored for all point-of-care tools. Each point-of-care tool was reviewed on both a smartphone and tablet of the different operating systems, as we only could get one score from each type of electronic device (n=4). The mean is the average total score for each device. The reviewer would then give a score of 0 or 1 to the different components of ease of use. The components are described in the Methods section.

As Table 2 depicts, DynaMedPlus had the highest score of ease of use (6.00), whereas Essential Evidence Plus was lowest (4.00), *P*<.01. The remaining four tools had medium scores with no significant difference (all *P*>.99).

### Quality Measure

Quality measures were scored for all point-of-care tools separately on each device, as we only could get one score from each type of electronic device (n=4). The mean is the average total score for each device. All point-of-care tools scored the same mean, with the exception of Medscape, which entirely lacked one of the components of the quality measure.

The reviewers rated same scores of quality (4.00) for DynaMed, DynaMedPlus, Epocrates, Essential Evidence Plus, and UpToDate (Table 3). Medscape had lower score of quality than others (*P*<.01).

**Table 1:** Breadth of coverage among six point-of-care tools.

Tool	n^a^	Mean	SD	LS-mean^b^	Multiple comparison	
Medscape	68	6.88	0.32	277.65	A	
DynaMedPlus	68	6.47	1.66	260.35	A	
Essential Evidence Plus	68	6.41	1.66	249.18	A	B
UpToDate	68	6.51	0.72	220.28		B
Epocrates	68	6.12	0.32	132.35		
DynaMed	68	5.53	1.26	87.19		

^a^Total number of diagnoses reviewed on all devices (17 diagnoses times 4 devices).

^b^Least square mean of rank score.

**Table 2:** Ease of use among six point-of-care tools.

Tool	n^a^	Mean	SD	LS-mean^b^	Multiple comparison	
DynaMedPlus	4	6.00	0.00	22.50		
DynaMed	4	5.00	0.00	13.00	A	
Epocrates	4	5.00	0.00	13.00	A	
UpToDate	4	5.00	0.00	13.00	A	
Medscape	4	4.75	0.50	10.50	A	
Essential Evidence Plus	4	4.00	0.00	3.00		

^a^Total number of devices.

^b^Least square mean of rank score.

**Table 3:** Quality among six point-of-care tools.

Tool	n^a^	Mean	SD	LS-mean^b^	Multiple comparison	
DynaMed	4	4.00	0.00	14.50	A	
DynaMedPlus	4	4.00	0.00	14.50	A	
Epocrates	4	4.00	0.00	14.50	A	
Essential Evidence Plus	4	4.00	0.00	14.50	A	
UpToDate	4	4.00	0.00	14.50	A	
Medscape	4	3.00	0.00	2.50		

^a^Total number of devices.

^b^Least square mean of rank score.

## Discussion

### Principal Findings

While studies exist that analyzed online point-of-care tools, this is the first study assessing access to the point-of-care tools on smartphones and tablet devices. As previous studies have shown, many online point-of-care tools are lacking in the breadth of coverage of medical topics, interface ease of use, and the quality of the contained contents [8,10-18]. The results from this study have been consistent with these findings, suggesting that medical personnel should not rely entirely on any single point-of-care tool.

The breadth of coverage analysis was designed to review the presented information within the point-of-care tool topic. Overall, there was no significant difference between the point-of-care tools for the criteria used to evaluate the breadth of coverage on common internal medicine topics because most point-of-care tools scored within the same range, with the exception of DynaMed. Clinicians usually refer to the point-of-care tools when seeking information on a clinical question and, while DynaMed provides excellent evidence-based information, it does not help with reasoning and arriving at an answer for the clinical question. The publishers may have realized this, addressing the issue with new product DynaMedPlus in 2015. This new product scored higher within the assessment of breadth of coverage, providing methods for reasoning through the care of a patient more than DynaMed.

The evaluation of ease of use showed the greatest variation in results among these tools. These criteria address the needs of the clinician’s experience, seeing how well these tools would be adapted into their workflow. All point-of-care tools are using appropriate methods like scrolling and using appropriate cues for linked items for easier mobile usage of the tools. The criteria of searching within a summary topic, as provided by UpToDate and DynaMedPlus, would be extremely useful to help clinicians easily locate what they are looking for during patient care. This feature would have been extremely helpful with Epocrates because it was more challenging to find an answer for each of the criteria defined in the breadth of coverage; there were many tabs to navigate to find the needed information.

Another component reviewed was the need for Internet connectivity to access the point-of-care tool content. This is an important criterion to consider as there are some clinicians with limited access to Wi-Fi or mobile data in their practice making it challenging to access care information at the time of need. Essential Evidence Plus, Epocrates, and UpToDate require a mobile data or Wi-Fi connection to access content, while Medscape, DynaMed, and DynaMedPlus download the summary topics to the device, needing data connection only for updates to the content. UpToDate offers an option for downloading its content on mobile devices with individual subscriptions for an additional charge.

All point-of-care tools also have the feature of creating a personal account; this allows for saving the user ID and password. All of the evaluated point-of-care tools have remote access through personal accounts and proxy institutional licenses, which is very convenient and beneficial.

The majority of the point-of-care tools were available using a native app, composed of pieces of software completely written in the native language of the mobile device platform such as Objective C for iOS and Java and C++ for Android [26]. The one exception in this study was Essential Evidence Plus, which uses a Web app modified for easy access on a mobile Web browser. The choice of development is dependent on a range of considerations when developing content for mobile devices [27]. The native apps integrate device-specific features (eg, GPS, camera) and provide off-line functioning but can be costly and time consuming to develop. The Web app allows for platform independence with use on any device by use of the Web programing language of HTML5 but can be limited in its features and speed. Overall, mobile point-of-care tool developers platform selection will be determined by optimizing the best user experience in the mobile arena.

All tools except Medscape cover all quality measure components. The missing component in Medscape was that it does not provide a date stamping of individual topic content at the time of the conducted research. While Medscape may have current content, the lack of date labeling of the content review or update can lead to questions of the information’s currency in a clinical application.

The researchers’ initial hypothesis was to not only test the point-of-care tools but to evaluate if there was any difference between the different devices. This study showed there was no difference based on the defined criteria between the mobile devices or operating systems. The use of a particular mobile device is highly subjective, based on user preferences of size, operating system, screen quality, cost, etc.

### Cost

Cost of the point-of-care tools will also influence the selection of the tools. The selected tools studied have differing price ranges for individual licenses depending on the training status of the purchaser. Among the six point-of-care tools, only Medscape is available at no charge.

As of December 2015, the following prices for the remaining five point-of-care tools were available via the product websites. DynaMed or DynaMedPlus costs US $99.95 per year for students, US $149.95 per year for residents, and US $395 per year for physicians. DynaMedPlus is freely available to members of the American College of Physicians at the time of this study. Essential Evidence Plus costs around US $85 per year, not distinguishing between the training status of the individual subscriber. Epocrates provides its drug content package for free; to have access to disease information and clinical practice guidelines, it costs US $174.99 per year for physicians and residents. UpToDate costs US $199 per year for students and residents and US $499 per year for physician. For use of the UpToDate mobile app, there is an additional charge of US $49 per year for mobile access of its content, and this access is restricted to two devices. With above-mentioned costs, all tools give full access to their contents.

Access to pricing information for institutional licensing was not available for inclusion in this study due to nondisclosure agreements.

### Limitations

Although the researchers attempted to build on evaluation tools used in the past, this study still has several limitations. The researchers were subject to the rapidly changing and inconsistent design environment of point-of-care tools. The creators/editors are constantly offering updates, so summary content and features may have been added after the dates of review.

Only six point-of-care tools were evaluated in the study; the researchers were limited in what was available via institutional licenses or individual subscription in the United States and what was accessible fitting the point-of-care tool selection criteria. This eliminated several of the previously evaluated point-of-care tools from this study, including BMJ Best Practices and PEPID.

During the interrater reliability measurement, there was a measured difference between the researchers due to a disagreement on the criteria defined for the “date of stamping for application platform.” This issue was reflected in the evaluation of the point-of-care tool ACP SmartMedicine, which was discontinued prior to the time of data collection for the full assessment. The definition was reviewed and a consensus was reached before the data collection for the full assessment.

ICD-9 codes selection focused on the specialty of medicine; it did not represent the broad spectrum of care thus limiting the assessment of comprehensiveness within the inpatient/outpatient settings. The breadth of coverage evaluation was impacted by the knowledge of the investigators to translate the given code to a synonym or related term to indicate the concept was covered within the point-of-care tool. The scoring system used was binary for all three areas of evaluation. Either the point-of-care tool contained the sought criteria or not; there was no gradient built within the assessment tool.

The investigators chose not to rank the point-of-care tools, as there is no scientific way to give appropriate weight to each of the components. Ranking would allow for personal bias to impact the weighting scale by considering one aspect of the evaluation criteria over another due to subjectivity.

### Conclusions

In evaluating the breadth of coverage, quality of information, and ease of use of six mobile point-of-care tools, the investigators were able to determine there is no significant difference between the point-of-care tools with regards to coverage on common topics used by clinicians within the discipline of medicine. The selection of a mobile point-of-care tool will likely depend on individual preference based on ease of use and cost of the application. For institutions subscribing to point-of-care tools via institutional licensing, it is important to gain the individual users’ perspective on selection of mobile point-of-care tools, because it enables choice of adoption by the main users of the product.

## Appendix

Multimedia Appendix 1 ICD-9 codes used to study breadth of coverage of point-of-care tools.

Multimedia Appendix 2 Breadth of coverage criteria and definitions.

Multimedia Appendix 3

Multimedia Appendix 4 Quality measures and definitions.

Multimedia Appendix 5 Ease of use factors and definitions.

## Abbreviations

HIMSS: Healthcare Information and Management Systems Society
ICD-9: International Classification of Diseases, Ninth Revision
mHealth: mobile health
