# Supplementary material for: Breadth of Coverage, Ease of Use, and Quality of Mobile Point-of-Care Tool Information Summaries: An Evaluation
Source: JMIR Mhealth Uhealth. 2016 Oct 12;4(4):e117. doi: 10.2196/mhealth.6189 (PMC5081478; doi:10.2196/mhealth.6189)
Supplement: Multimedia Appendix 1 [file mhealth_v4i4e117_app1.pdf]

Appendix 1: ICD-9 Codes Used to Study Breadth of Coverage of POCT

ICD-9 Code	Diagnosis
38.90	Septicemia, unspecified
410.71	Subendocardial infarction, initial episode of care
434.91	Cerebral artery occlusion, unspecified with cerebral infarction
584.90	Acute kidney failure, unspecified
491.21	Obstructive Chronic bronchitis with acute exacerbation
715.36	Osteoarthrosis, localized, not specified whether primary or secondary, lower leg
428.33	Acute on chronic diastolic heart failure
518.81	Acute respiratory failure
486.00	Pneumonia, organism not specified
427.31	Atrial fibrillation
272.40	Other and unspecified hyperlipidemia
401.90	Unspecified Hypertension
250.00	Diabetes mellitus Type II without complications not stated uncontrolled
327.23	Obstructive sleep apnea
244.90	Unspecified acquired Hypothyroidism
599.00	Urinary tract infection, site not specified
724.20	Lumbago
