# Supplementary material for: Breadth of Coverage, Ease of Use, and Quality of Mobile Point-of-Care Tool Information Summaries: An Evaluation
Source: JMIR Mhealth Uhealth. 2016 Oct 12;4(4):e117. doi: 10.2196/mhealth.6189 (PMC5081478; doi:10.2196/mhealth.6189)
Supplement: Multimedia Appendix 2 [file mhealth_v4i4e117_app2.pdf]

Appendix 2: Breadth of Coverage Criteria & Definitions

**Breadth of Coverage Criteria**	**Definition**	**Score**	
POCT provides a summary of the topic	Topic summary should define the most important items of information a physician would need for patient care	Absent 0	Present 1
Etiology	The science and study of the causes of disease and their mode of operation	Absent 0	Present 1
Patho-physiology	Derangement of function seen in disease	Absent 0	Present 1
Clinical Manifestations	The display or disclosure of characteristic signs or symptoms of an illness.	Absent 0	Present 1
Diagnosis	Determination of the nature of a disease, injury or congenital defect	Absent 0	Present 1
Treatment/Therapy	The treatment of disease or disorder by any method	Absent 0	Present 1
Prognosis	The forecast of the probably course and/or outcome of a disease.	Absent 0	Present 1

Definitions Source: Stedman’s Medical Dictionary
