# Supplementary material for: Breadth of Coverage, Ease of Use, and Quality of Mobile Point-of-Care Tool Information Summaries: An Evaluation
Source: JMIR Mhealth Uhealth. 2016 Oct 12;4(4):e117. doi: 10.2196/mhealth.6189 (PMC5081478; doi:10.2196/mhealth.6189)
Supplement: Multimedia Appendix 3 [file mhealth_v4i4e117_app3.pdf]

Appendix 3: Quality Measures & Definitions

Quality Measure	Definition	Score	
Inline references for treatment recommendations	References provided in-text, directly supporting individual statements within topic	Absent 0	Present 1
Inline references for diagnostic recommendations	References provided in-text, directly supporting individual statements within topic	Absent 0	Present 1
Date of stamping of individual content	Date of update provided at start or end of content provided	Absent 0	Present 1
Date of stamping for application platform	Date of update provided in footer of mobile app or in “About” content	Absent 0	Present 1
