# Supplementary material for: Breadth of Coverage, Ease of Use, and Quality of Mobile Point-of-Care Tool Information Summaries: An Evaluation
Source: JMIR Mhealth Uhealth. 2016 Oct 12;4(4):e117. doi: 10.2196/mhealth.6189 (PMC5081478; doi:10.2196/mhealth.6189)
Supplement: Multimedia Appendix 4 [file mhealth_v4i4e117_app4.pdf]

Appendix 4: Ease of Use Factors & Definitions

Ease of Use Factor	Definition	Score	
Search within summary content	User can search within topic to find information	Absent 0	Present 1
Table of Contents within topic	Topic contains appropriate Table of Contents for easy navigation	Absent 0	Present 1
Topic is displayed in format determined by best practices in mobile application design (Scrolling)	Appropriate scrolling patterns are utilized within the topic	Absent 0	Present 1
Topic is displayed in format determined by best practices in mobile application design (Affordance)	Making it clear what can be selected, tapped or swiped.	Absent 0	Present 1
Connectivity to internet	Mobile POCT need for Wi-Fi or mobile data connection for access to content	Yes 0	No 1
Personal account is easy to log into to access content	If personal account is required, they are easy to log into and modify settings for the POCT	Absent 0	Present 1
