# Supplementary material for: Breadth of Coverage, Ease of Use, and Quality of Mobile Point-of-Care Tool Information Summaries: An Evaluation
Source: JMIR Mhealth Uhealth. 2016 Oct 12;4(4):e117. doi: 10.2196/mhealth.6189 (PMC5081478; doi:10.2196/mhealth.6189)
Supplement: Multimedia Appendix 5 [file mhealth_v4i4e117_app5.pdf]

Multimedia Appendix 5: Calculation of General Linear Regression Model for the Breadth of Coverage, Ease of Use, and the Quality Measures for the comparison on the Mobile Point of Care Tools

Breadth of Coverage Measurement Score Rank

Tool	Score Rank
DynaMed	87.19
DynaMedPlus	260.35
Epocrates	132.35
Essential Evidence Plus	249.18
Medscape	277.65
UpToDate	220.28

Breadth of Coverage Least Squares Means for Effect Measures

	DynaMed	DynaMedPlus	Epocrates	Essential Evidence Plus	Medscape	UpToDate
DynaMed		<.0001	0.0026	<.0001	<.0001	<.0001
DynaMedPlus	<.0001		<.0001	1	1	0.0126
Epocrates	0.0026	<.0001		<.0001	<.0001	<.0001
Essential Evidence Plus	<.0001	1	<.0001		0.2591	0.2352
Medscape	<.0001	1	<.0001	0.2591		<.0001
UpToDate	<.0001	0.0126	<.0001	0.2352	<.0001	

Ease of Use Measurement Score Rank

Tool	Score Rank
DynaMed	13.0
DynaMedPlus	22.5
Epocrates	13.0
Essential Evidence Plus	3.0
Medscape	10.5
UpToDate	13.0

Ease of Use Least Squares Means for Effect Measures

	DynaMed	DynaMedPlus	Epocrates	Essential Evidence Plus	Medscape	UpToDate
DynaMed		0.0001	1	<.0001	1	1
DynaMedPlus	0.0001		0.0001	<.0001	<.0001	0.0001
Epocrates	1	0.0001		<.0001	1	1
Essential Evidence Plus	<.0001	<.0001	<.0001		0.0016	<.0001
Medscape	1	<.0001	1	0.0016		1
UpToDate	1	0.0001	1	<.0001	1	

Quality Measurement Score Rank

Tool	Score Rank
DynaMed	14.5
DynaMedPlus	14.5
Epocrates	14.5
Essential Evidence Plus	14.5
Medscape	2.5
UpToDate	14.5

Quality Least Squares Means for Effect Measures

	DynaMed	DynaMedPlus	Epocrates	Essential Evidence Plus	Medscape	UpToDate
DynaMed					<.0001	
DynaMedPlus					<.0001	
Epocrates					<.0001	
Essential Evidence Plus					<.0001	
Medscape	<.0001	<.0001	<.0001	<.0001		<.0001
UpToDate					<.0001	
